# During pregnancy, anticoagulant effect of low molecular weight heparin can be determined with both thrombelastography and anti-factor Xa activity, or not?

**DOI:** 10.1177/0310057X261453976

**Published:** 2026-07-10

**Authors:** Niclas Carlberg, Anna Olsson, Margareta Hellgren, Ove Karlsson

**Affiliations:** 1 Department of Obstetrics and Gynaecology, Sahlgrenska Academy University of Gothenburg, Gothenburg, Sweden; 2 Department of Anaesthesiology, Sahlgrenska University Hospital, Gothenburg, Sweden; 3 Department of Internal Medicine, Sahlgrenska Academy University of Gothenburg, Gothenburg, Sweden; 4 Department of Specialist Medicine, Sahlgrenska University Hospital, Gothenburg, Sweden; 5 Department of Obstetrics, Sahlgrenska University Hospital, Gothenburg, Sweden; 6 Department of Anaesthesiology and Intensive Care, Sahlgrenska Academy University of Gothenburg, Gothenburg, Sweden

**Keywords:** Anti-factor Xa activity, anti-FXa, dalteparin, low molecular weight heparin (LMWH), pregnancy, tinzaparin, thrombelastography (TEG), viscoelastic haemostatic assay (VHA)

## Abstract

Venous thromboembolism is a common cause of maternal mortality. Many women need regional anaesthesia during vaginal delivery or caesarean section. However, the risk of epidural or spinal haematoma is increased if regional anaesthesia is administered during ongoing thromboprophylaxis with low molecular weight heparin (LMWH). The aims of this pilot study are to evaluate the anticoagulant effect of LMWH during pregnancy by thrombelastography and anti-factor Xa (FXa) activity and their correlations. Sixteen pregnant women were administered dalteparin or tinzaparin from early pregnancy until six weeks postpartum. The anticoagulant effect was determined immediately before and three hours after injection. The target anti-FXa activity was 0.1–0.2 IU/ml pre-injection and less than 0.45 IU/ml post-injection. Thrombelastography variables were assessed at the same time. The correlations between anti-FXa activity and thrombelastography variables were modest, with correlation coefficients at 0.4–0.5. The thrombelastography variable best correlated to anti-FXa activity <0.1 IU/ml was thrombelastography K. The area under the receiver operating characteristic curve for thrombelastography K against anti-FXa activity <0.1 IU/ml was 0.77 (95% confidence intervals 0.64 to 0.91) with sensitivity 0.76 and specificity 0.81. The correlations increased as pregnancy progressed. The anticoagulant effect of LMWH can be measured either by analysis of anti-FXa activity or with thrombelastography. However, the correlation between the two methods is too low to allow replacement of one by the other. Further studies are necessary to determine which of the methods best predicts anticoagulant effect and increased risk of bleeding complications related to delivery or regional anaesthesia.

## Introduction

Venous thromboembolism (VTE) is one of the most important causes of maternal mortality in developed countries.^1,2^ The incidence of this obstetric complication is about 1 in 1000 pregnant women, with half of cases occurring during pregnancy and the other half postpartum.^3,4^ The incidence is increased four to five-fold compared with non-pregnant fertile-age women. The risk of VTE is further increased in patients with thrombophilia.^5^ The overall incidence can be reduced by thromboprophylaxis with anticoagulants. During pregnancy, this is administered as heparin or low molecular weight heparin (LMWH).^5,6^ Thromboprophylaxis increases the risk of spinal or epidural haematoma when administering regional anaesthesia at delivery.

Currently, about 50% of the pregnant women in Sweden have epidural analgesia (EDA) for pain relief at delivery. Further, regional anaesthesia is also the first choice of anaesthesia at caesarean section in order to minimise severe complications.^7,8^ However, regional anaesthesia can lead to epidural or spinal haematoma if coagulation is impaired.^9^ Thus, providing effective thromboprophylaxis around delivery without increasing the number of women denied EDA/spinal due to increased risk, can be problematic. Viscoelastic haemostatic assays such as thrombelastography (TEG) and thromboelastometry (ROTEM), are used to investigate haemostasis in connection with bleeding complications related to obstetric clinical conditions.^10–12^ However, few published studies have investigated these methods for determining the anticoagulant effect of LMWH during pregnancy.^13–16^ More pregnant women with anticoagulant treatment could probably be allowed EDA/spinal at delivery if reliable bedside analysis of the anticoagulant effect were quickly available.

The aims of this pilot study are to evaluate the anticoagulant effect of LMWH during pregnancy by TEG and anti-factor Xa (FXa) activity and their correlations.

## Materials and methods

### Study population

The study was performed at the Department of Obstetrics, Sahlgrenska University Hospital, Gothenburg, Sweden. Patients were included during two periods 2005–2006 and 2011–2012. The participating women were being treated with high-dose thromboprophylaxis, due to very high risk of VTE, according to the national guidelines in Sweden.^17^ Indications included thrombophilia, hereditary conditions, and history of recurrent VTE, massive thrombosis or pulmonary embolism (Table 1). The study was approved on 6 December 2004 by the Swedish Ethical Review Board (approval number 306–04) and the women gave written informed consent.

**Table 1.** Demographics, previous venous thromboembolism and thrombophilia.

Demographics	Dalteparin, *n* = 10	Tinzaparin, *n* = 6
Age, years, mean (SD)	31.7 (5.7)	30.8 (3.2)
BMI, mean (SD), early first trimester	27.9 (7.3)	23.7 (5.9)
Parity, no. of patients		
Nullipara	2	3
Multipara	8	3
Previous venous thromboembolism, n		
Deep vein thrombosis	9	4
Pulmonary embolism	0	2
Thrombophilia, *n*		
FV Leiden, heterozygous	2	1
FV Leiden, homozygous	1	0
FV Leiden and protein C deficiency	1	1
Combined FV and FII mutation	1	1
Protein S hereditary deficiency	0	1
High FXI (hereditary)	1	0
High FVIII (hereditary)	0	1
None	4	1

*n*: number; FV: factor V; FII: factor II prothrombin gene mutation; FXI: factor XI; FVIII: factor VIII; BMI: body mass index (kg/m^2^); SD: standard deviation.

### Anticoagulant treatment

Thromboprophylaxis with LMWHs dalteparin or tinzaparin was started as soon as possible in the first or early second trimester and administered twice daily. The two different medications were due to changed regimens at the hospital where the study took place. Doses were adjusted based on anti-FXa activity, according to the respective regimen. The anticoagulant effect was routinely determined, immediately before and 3 hours post-injection. This was assessed monthly, or weekly if the dose was changed. The aim of the thromboprophylaxis was to achieve measurable 24 hour anticoagulant effect. Blood sampling and comparison of anticoagulant effect took place at least at gestational week (gw) 10–15, 20–22, 28–30 and 38–40. The target anti-FXa activity was 0.1–0.2 IU/ml before and less than 0.45 IU/ml 3 hours post-injection. During delivery, the dose was decreased to 2500 U dalteparin or tinzaparin every 12 hours. Postpartum, thromboprophylaxis with anticoagulant efficacy equalling that during pregnancy was continued for at least 6 weeks.

### Blood sampling and laboratory methods

Blood sampling via direct venepuncture, was performed between 08:00 and 12:00 hours by nurses trained in the TEG method. Whole blood for anti-FXa activity analysis was added to 0.13 M citrate. Within 60 minutes, it was centrifuged for 20 minutes at 2000 ***g***. Plasma was analysed immediately or kept frozen at −20°C until analysed. Anti-FXa activity was determined with chromogenic substrate, STA-Rotachrom or STA-Liquid (Diagnostica Stago SAS, France), without addition of antithrombin. The normal reference value in the absence of thromboprophylaxis was <0.1 IU/ml during most of the study. The anticoagulant effect of LMWH was regarded as unmeasurable at values under this reference level. During a period at the beginning of the study, the corresponding reference value was <0.05 IU/ml.

The effect of LMWH on the viscoelastic method TEG was analysed using TEG 5000 version 4.2 software (Haemoscope Corporation, Niles, IL, USA). Whole blood for TEG was sampled at the same time as samples were taken for anti-FXa activity. One millilitre of whole blood was added to containers prepared with kaolin or kaolin-heparinase, respectively, and 0.36 ml from each sample was used for analysis. TEG analysis was performed within 4 minutes of sampling and run for 90 minutes. The following TEG variables were assessed: time until start of clotting (TEG-R), time until 20-mm clot firmness (TEG-K), angle of clotting (TEG-Angle), maximum amplitude (TEG-MA), lysis after 30 minutes (TEG-LY30), Delta-R (TEG-R (kaolin) minus TEG-R (kaolin and heparinase)) and thrombodynamic ratio (TDR).

### Statistics

Continuous variables are presented with mean (standard deviation, SD) and median (range), while categorical variables are presented with *n* (%). Correlations between anti-FXa activity and TEG variables were calculated using Spearman’s correlation, in two different ways. First, the correlation between these variables was calculated for each patient based on all available measurements for the individual patient. The mean correlations for each variable and patient were then calculated and used to generate the final mean correlation coefficients for each TEG variable and the 16 patients. These mean correlation coefficients from the 16 patients were then tested with the Wilcoxon signed-rank test if they were separate from zero. Second, Spearman correlation coefficients were calculated for each gw, based on available results at each timepoint.

Logistic regression was applied to determine the best cut-off values of TEG variables to predict anti-FXa activity <0.1 IU/ml, with TEG variable as an independent variable. A receiver operator characteristic (ROC) curve analysis was performed to identify the optimal risk cut-off for TEG variables, minimising the distance from the point of the ROC curve to optimum point (0, 1). All the tests were two-tailed and conducted at the 5% significance level. All analyses were performed using SASv9.4 (SAS-Institute, Cary, NC, USA).

## Results

Sixteen pregnant women with very high risk of VTE, administered high dose thromboprophylaxis with LMWH from early pregnancy until at least 6 weeks postpartum, were included. Dalteparin was administered to 10 women and tinzaparin to six women. Demographic factors, previous VTE and risk factors are presented in Table 1.

### Anticoagulant treatment

**Figure 1.**
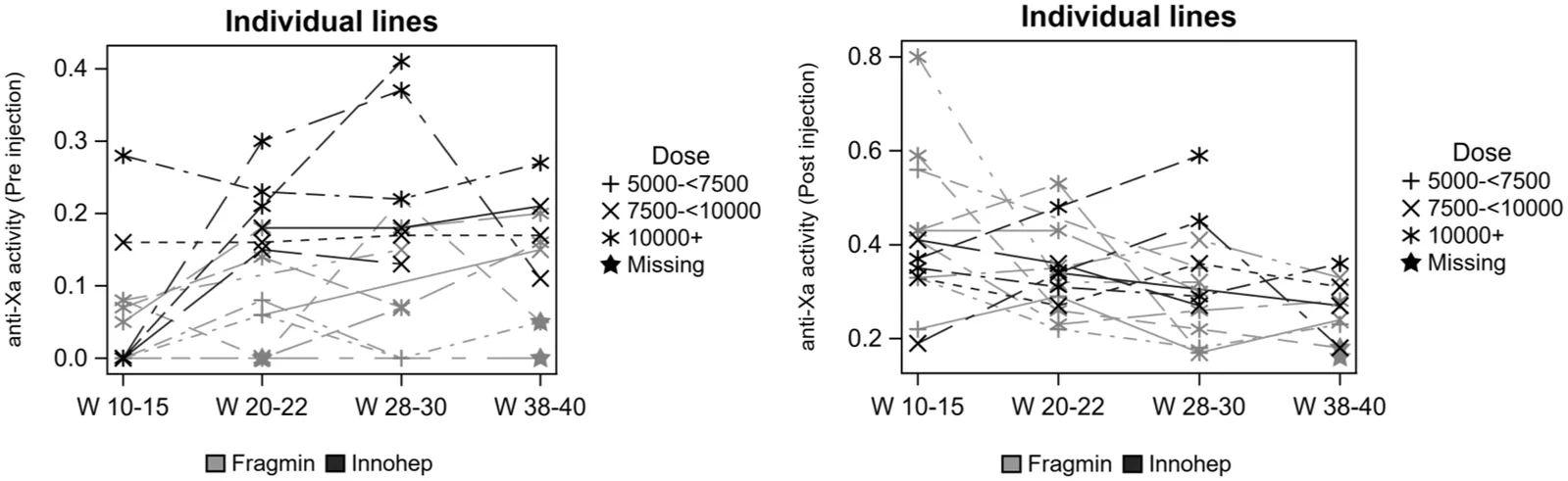
Anti-factor Xa activity at different gestational weeks, pre-injection and post-injection.

**Figure 2.**
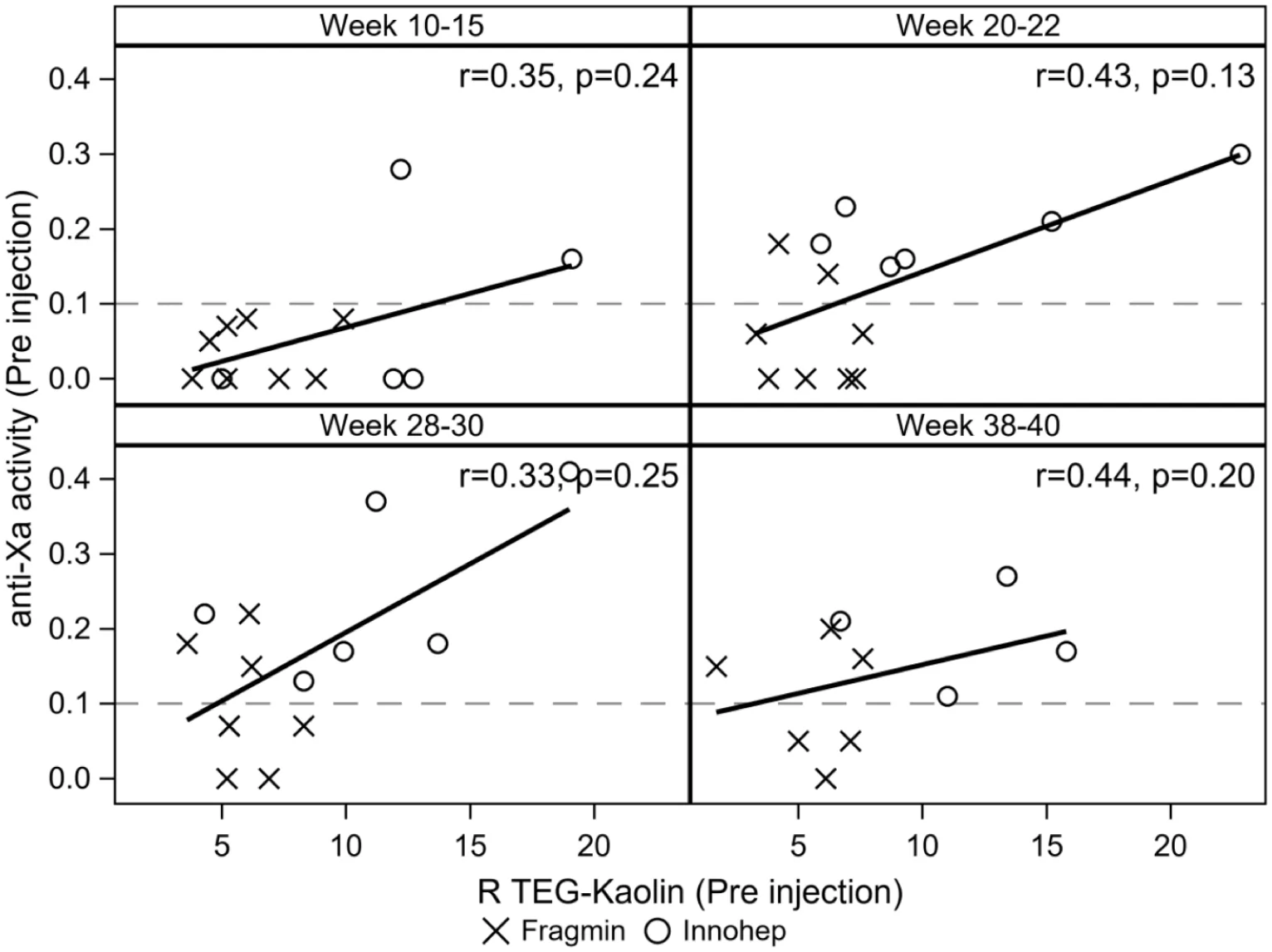
Pre-injection anti-factor Xa activity and TEG-R during all trimesters. Week: gestational week.

The anticoagulant effect of LMWH was mainly within the target limits of anti-FXa activity throughout pregnancy (Figure 1). TEG variables seemed to be more affected during tinzaparin than during dalteparin treatment, as exemplified by TEG-R in Figure 2. Overall correlations between anti-FXa activity and TEG-R, TEG-K, TEG-Angle and Delta-R are shown in Table 2. The correlations between the two methods (anti-FXa activity and TEG) varied during pregnancy. The strongest correlations were seen in the pre-injection samples from gw 22 to term, measured by TEG-K and TEG-Angle, and in the post-injection samples during gw 20–22 measured by TEG-R (see Table 3).

**Table 2.** Correlation between anti-factor Xa activity and thrombelastogram variables without adjustment for gestational week, presented for total population and for the respective low molecular weight heparin.

TEG variables	All		Dalteparin		Tinzaparin	
	Mean (SD)	*P* within group	Mean (SD)	*P* within group	Mean (SD)	*P* within group
	Median (Min; Max)		Median (Min; Max)		Median (Min; Max)	
	(95% CI)		(95% CI)		(95% CI)	
	*n* = 16		*n* = 10		*n* = 6	
TEG-R	0.460 (0.397)	0.0010	0.426 (0.412)	0.020	0.518 (0.400)	0.063
	0.555 (−0.467; 0.943)		0.46 (−0.467; 0.886)		0.598 (−0.154; 0.943)	
	(0.249 to 0.672)		(0.131 to 0.721)		(0.098 to 0.938)	
TEG-K	0.445 (0.338)	0.0002	0.471 (0.344)	0.0039	0.403 (0.354)	0.063
	0.497 (−0.103; 0.866)		0.512 (−0.029; 0.866)		0.402 (−0.103; 0.802)	
	(0.265 to 0.625)		(0.224 to 0.717)		(0.031 to 0.774)	
TEG-Angle	−0.495 (0.314)	<0.0001	−0.435 (0.316)	0.0039	−0.596 (0.311)	0.031
	−0.514 (−0.943; 0)		−0.368 (−0.873; 0)		−0.645 (−0.943; −0.079)	
	(−0.663 to −0.328)		(−0.661 to −0.209)		(−0.922 to −0.270)	
Delta-R	0.536 (0.271)	<0.0001	0.527 (0.297)	0.0020	0.551 (0.249)	0.031
	0.518 (0.036; 1)		0.459 (0.036; 1)		0.582 (0.205; 0.886)	
	(0.391 to 0.680)		(0.315 to 0.739)		(0.289 to 0.812)	

Spearman’s correlation was used to calculate the correlations for all variables.

Wilcoxon’s signed-rank test was used for comparison within groups.

CI: confidence intervals; TEG: thrombelastogram.

**Table 3.** Correlations between anti-factor Xa activity and thrombelastogram variables at different gestational weeks and pre or post-injection.

TEG variables (kaolin)		Gestational week and relation to LMWH injection							
		10–15	10–15	20–22	20–22	28–30	28–30	38–40	38–40
		Pre	Post	Pre	Post	Pre	Post	Pre	Post
TEG-R	*r*	0.35	−0.04	0.43	**0.56**	0.33	0.30	0.44	0.22
	*P*	0.24	0.90	0.13	**0.037**	0.25	0.32	0.20	0.54
	*n*	13	14	14	**14**	14	13	10	10
TEG-K	*r*	0.38	0.02	**0.55**	0.51	**0.61**	0.31	**0.71**	0.33
	*P*	0.20	0.96	**0.042**	0.062	**0.021**	0.30	**0.021**	0.36
	*n*	13	14	**14**	14	**14**	13	**10**	10
TEG-Angle	*r*	−0.25	−0.09	−0.42	**−**0.51	**−0.58**	−0.38	**−0.75**	−0.36
	*P*	0.42	0.75	0.13	0.063	**0.028**	0.20	**0.012**	0.31
	*n*	13	14	14	14	**14**	13	**10**	10
Delta-R	*r*	0.30	−0.13	0.29	0.36	0.39	0.37	0.54	0.59
	*P*	0.33	0.66	0.32	0.20	0.17	0.24	0.10	0.12
	*n*	13	13	14	14	14	12	10	8

Pre: pre-injection; post: 3 hours post-injection; *r*: correlation coefficient; *P: P*-value; *n*: number; Delta-R: TEG-R (kaolin) – TEG-R (kaolin + heparinase).

Bold type: S﻿ignificant correlations. The Spearman’s correlation with corresponding *P*-value and number of presentations is presented for all variables.

No adjustments for different low molecular weight heparins (LMWHs).

**Figure 3.**
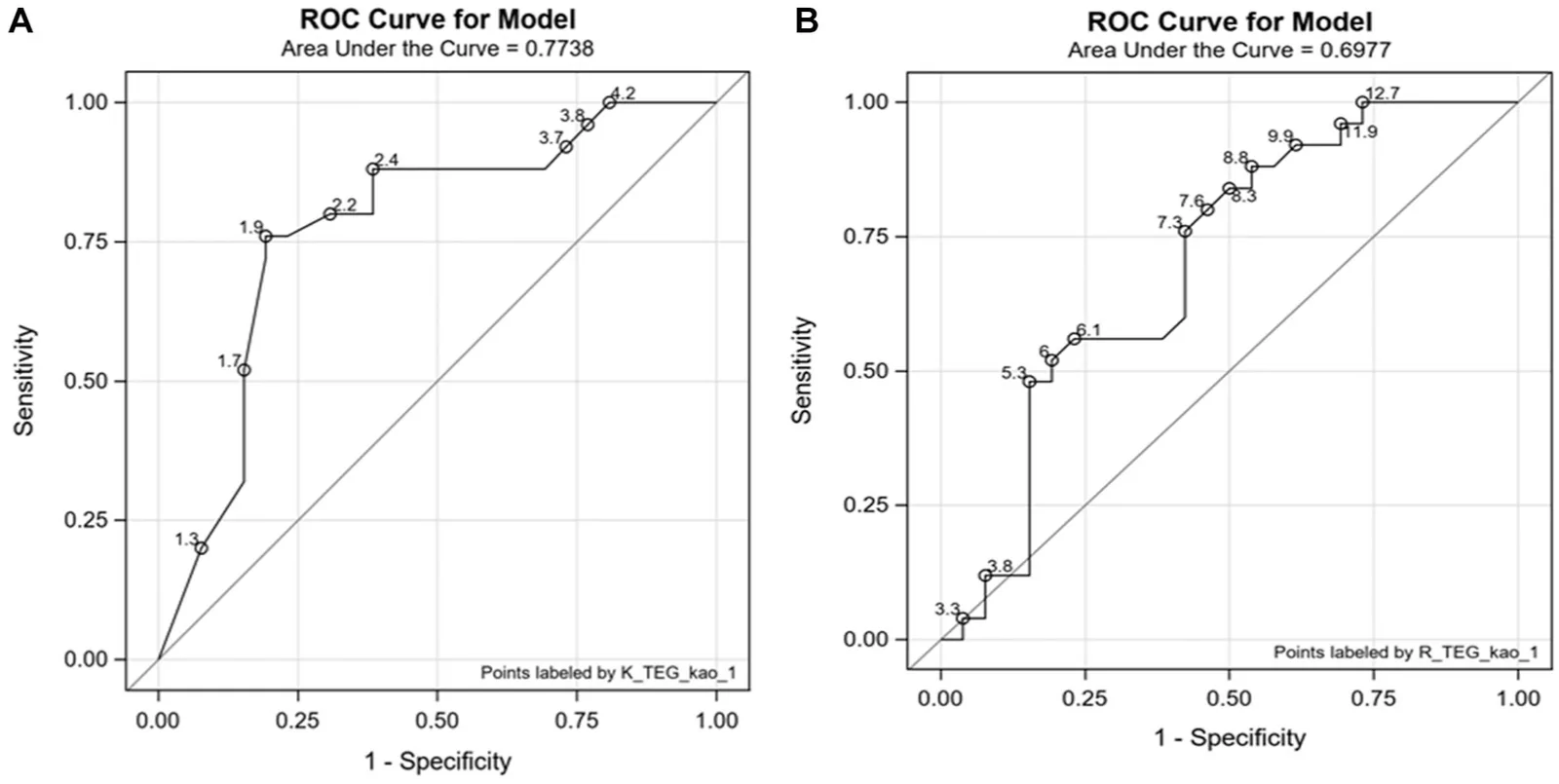
Area under the curve for (a) TEG-K and (b) TEG-R against anti-factor Xa activity < 0.1 IU/ml. No adjustment for gestational weeks or different LMWHs.

The TEG variable best correlated to anti-FXa activity <0.1 IU/ml was TEG-K. The area under the ROC curve for TEG-K against anti-FXa activity <0.1 IU/ml was 0.77 (95% confidence intervals (CI) 0.64 to 0.91). Sensitivity was 0.76 and specificity was 0.81. The area under the ROC curve for TEG-R against anti-FXa activity <0.1 IU/ml was 0.70 (95% CI 0.55 to 0.85). Sensitivity was 0.76 and specificity was 0.58 (Figure 3). TEG-MA, TEG-LY30 and TDR correlated more weakly to anti-FXa activity than the other TEG variables.

### Clinical outcomes

Clinical outcomes are presented in Table 4. There was no VTE recurrence during pregnancy. One woman contracted superficial thrombophlebitis 3 weeks postpartum, despite ongoing thromboprophylaxis. Her anti-FXa levels 8 weeks postpartum were 0.34 IU/ml pre-injection and 0.47 IU/ml post-injection. She had had deep vein thrombosis (DVT) several times, beginning at 14 years of age. Protein S deficiency had been diagnosed before the current pregnancy and the maximal dose of tinzaparin given during pregnancy was 14,500 IU per 24 hours. She had two relatives with a history of DVT.

**Table 4.** Clinical maternal and neonatal outcomes.

Maternal outcome	Dalteparin	Tinzaparin
Recurrence of VTE		
• During pregnancy	0	0
• Postpartum	0	One superficial thrombophlebitis
Bleeding complications during pregnancy	Two minor vaginal bleeds at onset of delivery	One minor bleed early in pregnancy
Estimated bleeding at delivery, ml, mean (SD)	700 (433)	640 (354)
Bleeding complications at delivery/postpartum	One premature placental abruption* One uterine atony	One abdominal wall bleeding One uterine atony
Neonatal outcome		
Status	All but two healthy Two SGA and premature (twins)*	All healthy
Birth weight, g, mean (SD)	3467 (672)	3487 (433)

VTE: venous thromboembolism; *: same pregnancy; SGA: small for gestational age; g: grams; SD: standard deviation.

There was one minor incident of vaginal bleeding in early pregnancy and one premature placental abruption in a woman with previous jugular vein thrombosis. There were two minor incidents of vaginal bleeding at onset of delivery, none of which were assessed as being due to anticoagulants. Nine women had spontaneous vaginal deliveries. There were three inductions of labour and two planned and two emergency caesarean deliveries. All but two babies were healthy. Two (twins) were premature and small for gestational age. They were delivered due to premature placental abruption, resulting in an emergency caesarean section. No EDA was administered, and the sole spinal anaesthesia was administered for a planned caesarean section. The increase in maternal body weight during pregnancy ranged from 6 to 18 kg.

## Discussion

Overall, there were modest correlations between TEG variables and anti-FXa activity in our cohort of women with ongoing obstetric thromboprophylaxis with LMWH. However, correlations increased as pregnancy progressed but were too weak to allow replacement of anti-FXa activity analysis with TEG for determination of anticoagulant effect. Clinicians should not determine the safety of regional anaesthesia in pregnant women treated with LMWH based on a TEG result until more knowledge is available.

Currently, the chromogenic anti-FXa assay is regarded as the gold standard for monitoring LMWH therapy.^18^ The two methods investigated here measure anticoagulant effect in different ways. Anti-FXa activity determines anticoagulant effect of LMWH through the inhibition of factor Xa in citrate plasma and is performed in the laboratory and seldom acutely available.^19^ TEG, on the other hand, presents the overall inhibitory effect on whole blood coagulation and is a point-of-care method giving a result in 10–15 minutes. LMWHs primarily inhibit FXa, but they also inhibit factor IIa, to an extent depending on the specific LMWH. This does not affect the results of the anti-FXa assay, but may influence the potency of different LMWHs and thus the total anticoagulant effect.^20^ The anti-FXa assay used in this study was performed without added exogenous antithrombin, to ensure that the in vitro coagulation results emulated in vivo coagulation.^21^ The relation between anti-FXa activity, TEG and risk of bleeding is not entirely clear. It might be assumed that TEG is more sensitive for different types of LMWH, especially those with higher molecular weight and greater anti-factor II effect. Theoretically, TEG would be a better alternative than anti-FXa activity to exclude the risk of bleeding complications, but this must be proved in future studies.

The results of our study are not consistent with prior studies comparing anti-FXa activity with TEG for monitoring LMWH effect during pregnancy.^14,15^ Carroll et al.^14^ reported a strong correlation between anti-FXa activity and Delta-R in obstetric patients throughout pregnancy and at term. An in vitro pilot study confirms these findings, presenting high corresponding sensitivity and specificity for TEG-R and TEG-K. Plasma was then sampled from pregnant women and LMWH was added to plasma in vitro. Non-anticoagulated samples and those with LMWH added in vitro were compared.^15^ Conflicting results concerning correlations between TEG parameters and anti-FXa activity, for evaluation of LMWH anticoagulant effect, have been reported in other clinical settings and in healthy volunteers.^13,22,23^ Such comparisons are actually not advisable, because the TEG variables normally show increasing coagulability during pregnancy, which may influence the results.^24^ We have shown earlier how TEG variables change in a healthy cohort during normal pregnancy and to 8 weeks postpartum.^24^ The increasing correlation as pregnancy progresses may be due to physiological changes in the coagulation but this study does not answer that.

TEG and ROTEM are useful point-of-care methods in the care of patients with obstetric complications, trauma, major bleeding or impaired haemostasis, especially regarding the result within 10–15 minutes.^25^ Scientific evidence has increased in recent years and these point-of-care methods provide good guidance in the care of patients with severely affected haemostasis. It would be helpful to determine the anticoagulant effect when delivery patients with ongoing thromboprophylaxis request regional analgesia. Pavoni et al.^26^ reported varying results regarding ROTEM parameters. This review shows modest to weak correlations between anticoagulant effect and ROTEM parameters.

The efficacy of thromboprophylaxis, reflected as recurrence of VTE, was good. Superficial thrombophlebitis postpartum in the woman with hereditary protein S deficiency might have been due to underdosed LMWH. The appropriate dose is not known, but women with protein C or S deficiency are at especially high risk of VTE during the puerperium, as has been reported previously.^5,17,27^

There were minor bleeding complications during pregnancy, probably not due to LMWH. This has been reported by other authors.^5,6^ The quite substantial bleeding at delivery in some patients in our study might have been due to excessive anticoagulant dosage during delivery or to late dose reduction during the weeks immediately preceding delivery. Current guidelines recommend withdrawal of LMWH at labour onset.^5,27–29^ However, administering some kind of anticoagulant can be necessary in very high risk cases. We have long applied the current regimen, shown in the Methods section of this paper, without serious complications. The most probable explanations for bleeding at delivery are excessive LMWH dose before onset of labour and obstetric causes such as uterine atony. The appropriate dose is not yet known.

To our knowledge, this is the only study comparing anti-FXa activity and TEG analyses during ongoing obstetric thromboprophylaxis with LMWH in clinical conditions. Although the study population is rather small, a large number of blood samples were taken during different periods of pregnancy. Furthermore, blood sampling and analyses were carried out during clinically appropriate conditions.

The sample size is small, which limits the generalisability. The study population consisted of women administered thromboprophylaxis with one of two LMWHs, chosen according to clinical routines. The two LMWHs could not be compared due to small group size. True randomisation and a larger study population would have been better and would have enabled this comparison. The number of women recruited was due to the decision to perform a pilot study. A larger study is needed to verify our results and to determine which method best detects bleeding risk during pregnancy and delivery in cases with ongoing LMWH thromboprophylaxis. Epidural or spinal bleeding is a rare complication^9^ and would not have been expected in our population, so the risk of this potentially serious event must be assessed by the proxy of present gold standard, i.e. anti-FXa activity.

## Conclusions

The anticoagulant effect of LMWHs dalteparin or tinzaparin can be measured either by analysis of anti-FXa activity or with the viscoelastic method TEG. However, the correlation between the two methods is too low to allow replacement of one by the other. Further larger studies are necessary to determine which of the methods best predicts anticoagulant effect and increased risk of bleeding complications related to delivery or regional anaesthesia.
